# Analyzing the Effect of Government Subsidy on the Development of the Remanufacturing Industry

**DOI:** 10.3390/ijerph17103550

**Published:** 2020-05-19

**Authors:** Qin Zhou, Kum Fai Yuen

**Affiliations:** School of Civil and Environmental Engineering, Nanyang Technological University, Singapore 639798, Singapore; zh0031in@e.ntu.edu.sg

**Keywords:** circular economy, trade old for remanufactured, government subsidy, budget constraint, network externality

## Abstract

Remanufacturing plays an important role in a circular economy, by shifting supply chains from linear to closed loop. However, the development of the remanufacturing industry faces many challenges. Consumers’ uncertainty about the quality of remanufactured products can hamper their decision to make a purchase (i.e., uncertainty behavior). Such uncertainty can be reduced when they learn that more consumers are purchasing remanufactured products (i.e., network externality behavior). Considering the aforementioned behaviors, this paper investigates how a government could set the optimal subsidy level to maximize the sales quantity of remanufactured products with a limited budget. We modeled a Stackelberg game between the government and an original equipment manufacturer, under two settings, over two periods. Setting 1 only considers an original equipment manufacturer that produces remanufactured products, and Setting 2 considers an original equipment manufacturer that produces both new and remanufactured products. We show that the original equipment manufacturer should adjust its pricing strategy (i.e., markup vs. markdown) according to the subsidy levels. Our analysis on the government budget constraint shows that an original equipment manufacturer earns more profits in Setting 1 than Setting 2, only when the budget constraint is high, and less profits when budget constraint is low.

## 1. Introduction

Developing a circular economy is gaining significant momentum worldwide, with the aim of shifting supply chains from linear to closed loop [1]. Firms are encouraged by environmental groups and authorities to embed material circularities within supply chains, and this can include reusing, repairing, refurbishing, remanufacturing, and recycling [2]. Among these circularities, remanufacturing has played an increasingly significant role, because it can deliver all-round sustainability benefits. On the one hand, remanufacturing presents a natural low-cost alternative to traditional manufacturing, because it can exploit the residual value of used products. Many industry practices have proved that remanufacturing can be a highly profitable business [3,4,5]. For instance, Kodak, BMW, IBM, DEC, Xerox, Hewlett Packard Corporation, and Canon have all benefited from their remanufacturing operations, either by saving on material costs or selling remanufactured products. In addition, remanufacturing can significantly alleviate environmental burden by decreasing the demands for natural resources and waste production. Remanufactured products can prolong the useful life of materials, which can reduce landfill space and carbon emission [6,7]. However, remanufacturing is usually faced with several challenges. 

The first challenge is associated with the development of the remanufacturing industry. In many countries, both formal and informal recycling sectors exist [8]. The problem faced by the informal sector is that recyclers’ dissembling and disposal methods are often rudimentary and do not receive proper qualifications from the government. This reduces the effectiveness of any recycling or remanufacturing effort. The problem faced by the formal sector is that it has an apparent disadvantage in disposing cost, which poses difficulty for its firms to provide a competitive acquisition price [8]. The second challenge concerns consumers’ acceptance and willingness to pay for remanufactured products. Existing studies suggest that consumers will buy remanufactured products if sufficient discounts are offered [9,10]. In some cases, even with discounts, consumers would not consider buying remanufactured products, because of their uncertainty about the quality of remanufactured products [7,11,12]. 

The problems linked with recycling and consumers’ uncertainty about the quality of the remanufactured products can hinder the progression of closed-loop supply chains and the remanufacturing industries. To regulate collection and reduce consumers’ uncertainty, subsidy programs have been introduced. For instance, in 2013, the Chinese government launched a subsidy program that was called “trade old for remanufactured” (TOR) [13]. The government selected 10 automakers or engine manufacturers as the pilot firms to execute the TOR program [14]. Consumers can trade-in their old product for a remanufactured product, under this program, and obtain a subsidy. The goal of the government subsidy is to encourage consumers with uncertainty to purchase remanufactured products and increase collection volume for remanufacturing firms, which can help these firms better cope with challenges they face. For instance, a government subsidy can increase participating firms’ quality of old products because consumers have to provide accurate information about their trade-in product, which can in turn improve the quality of remanufactured product and ease consumers’ uncertainty. The subsidy can also improve the market share of remanufacturing firms by directly offering a subsidy to consumers and thus induce the consumers to make a purchase. Despite the benefits that a government subsidy can offer to the development of a circular economy, the following question remains unanswered: How should the government set the subsidy to maximize the sales quantity of remanufactured products with a limited budget?

Compared to previous studies, this study builds on the premise that a government subsidy program can encourage more consumers to buy remanufactured products. In fact, when consumers are uncertain about the quality of the products, they are reluctant to make a purchase. However, their uncertainty can be eased if more consumers buy the products [15]. This behavior can be explained by network externality theory. Network externality states that consumers’ behavior is affected by the behavior of others, and consumers’ utility increases with the number of other consumers consuming the product [15]. The government’s subsidy can have mixed effects on the consumers, as the government can have budget constraints and may only provide the subsidy in one period. On the one hand, consumers would more prefer to wait until other consumers purchase the product, so that their uncertainty can be greatly eased. On the other hand, the subsidy encourages consumers to purchase early to obtain the subsidy. The mixed effects make consumers’ purchasing behavior more complex than usual and pose greater challenges for firms serving these consumers.

The originality of this study lies in the incorporation of the aforementioned consumers’ behavior into the model and the analysis of the effect of the behavior on firms’ pricing decision and the effectiveness of a government subsidy. This study attempts to address the following research questions: How does consumers’ uncertainty affect remanufacturing firms’ optimal pricing decisions? How does subsidy affect the development of the remanufacturing industry? What should be the reasonable level of subsidy provided by the government? 

The existing research on remanufacturing and collection mainly focuses on choosing the optimal channel for manufacturer, designing reverse logistics network, managing remanufacturing, and coordinating new and remanufactured products in the same market. Despite the fact that many remanufacturing firms have limited access to used products for recycling and consumers’ reluctance to purchase remanufactured products due to uncertainty, very few studies have focused on investigating the effectiveness of a government subsidy on consumers’ purchase behavior and regulating recycling. The objective of this study is to fill the gap and assist government to promote remanufacturing and regulate the recycling industry toward a circular economy. 

The paper is organized as follows. In the next section, we briefly review the previous work. In Section 3, we introduce the model description and setup. In Section 4, we present the main model and analysis. In Section 5, we provide additional discussion of the government budget constraint. We discuss the managerial implications in Section 6. Finally, Section 7 contains the concluding remarks. All proofs are provided in the Appendix A.

## 2. Literature Review

In this section, we review several streams of literature that relate to our research. The streams of literature include the following: (a) circular economy or sustainable supply chain; (b) government subsidy; (c) network externality; (d) consumers’ perceptions of remanufactured products; and (e) coexistence of new and remanufactured products. 

Our work is closely related to the circular economy or sustainable supply chain. Circular economy is an economic paradigm which involves extracting maximum value from natural resources and prolonging their use for as long as possible. Rooted in industrial ecology, the emphasis of this paradigm is to recycle waste materials, extending the boundary of sustainable supply chain management [16]. Over the last two decades, sustainable supply chain management has received significant interest, particularly in operations management (see [1] for a comprehensive review). Some researchers have investigated the adoption of new models to improve supply chain sustainability. For instance, Agrawal and Ülkü [17] investigated the conditions where modular upgradability can benefit the environment and firms. Then, Agrawal and Bellos [18] examined the economic and environmental potential of an innovative business model, that is, firms can sell the utility of a product instead of the product itself. Guo et al. [19] showed that the overall supply chain responsibility can be improved by focusing on consumers or the whole supply chain. Alizadeh-Basban and Taleizadeh [20] employed three game theoretical approaches for a dual supply chain toward a circular economy. Another stream of literature studies the impact of environmental regulation on supply chain sustainable development. Miao et al. [21] found that the implementation of carbon regulations can promote the development of the remanufacturing industry but can decrease a manufacturer’s profits. Alev et al. [22] investigated the enactment of extended producer responsibility legislation on durable and nondurable products and found that the implementation parameters, with respect to the two products, should differ. Consistent with this stream of literature, we also consider the sustainable development of supply chain. More specifically, we investigate the role of the government in promoting the sustainable development of remanufacturing. 

Some researchers discuss the effect of a subsidy on improving sales quantity. Lobel and Perakis [23] determined the optimal subsidies for policymakers who want to achieve a desired solar technology adoption level with minimum cost. They also considered consumers who may imitate other consumers’ behavior in the new technology adoption process. Krass et al. [24] examined how environmental taxation with fixed cost subsidies affects firms’ green technology choice. Xiao [25] investigated the distribution, purchase, or the sale channel that the government should subsidize in order to improve consumers’ access to certain products, such as malaria drugs. Cohen et al. [26] studied the impact of demand uncertainty on the policy design of various players (e.g., government, industry, and consumers) and the influence of a government subsidy to accelerate the adoption of a new technology. Levi et al. [27] examined the effectiveness of uniform subsidies in increasing goods’ consumption when facing budget constrain and uncertain market response. Yu et al. [28] investigated the allocation of a government subsidy to improve consumer welfare. Yu et al. [29] addressed several problems for donors in a supply chain when they want to subsidize products for low-income families. Their study analyzed the issue of whom to subsidize and the extent of the subsidy. Zhu et al. [30] compared two different methods (i.e., cash subsidy and imposing carbon regulation) on promoting the remanufacturing industry and noted that they have different effects on remanufacturing. Cao et al. [31] showed that the government’s new policies and technical support greatly improved the remanufacturing industries’ industrial scale and annual production value. Hu et al. [32] examined two government subsidy strategies, direct subsidy and policy bias, and the two strategies were both helpful for the development of remanufacturing industry. Motivated by the Chinese government’s TOR program, we investigated the optimal allocation of a government subsidy to consumers, for the purchase of remanufactured products, and the extent of the subsidy with and without a budget constraint. Further considering consumers’ behavior, we also considered network externality by examining the effect of the subsidy on consumers’ purchase behavior, by considering the influence of earlier adopters. 

Network externality is a crucial theory in our model. The concept of network externality has its roots from networked goods (e.g., communication technologies), which later branched out to other types of goods [33]. Externalities can be classified into positive and negative, depending on their impact on consumers’ consumption. Positive externality posits that consumers’ utility increases as more consumers purchase the product. For instance, Candogan et al. [34] investigated a monopolist’s optimal pricing strategies, which consider positive network effects. Negative externality describes the situation that consumers’ utility decreases as more consumers buy the product. Such an example can occur in the transportation or luxury industry. In the transportation industry, as more drivers choose the same route, possible congestions would occur. Meanwhile, in the luxury industry, the negative effect mainly comes from consumers’ seeking exclusivity or status behavior. As such, consumers’ consumption utility decreases as the number of buyers increases [35,36]. Our research relates to positive externality. This applies to remanufactured products, where consumers feel uncertain about the quality of the remanufactured product and are reluctant to make a purchase. Consequently, more consumers will purchase the remanufactured product when the government provides a subsidy to consumers and when many other consumers purchase the product, thereby reducing consumers’ uncertainty. 

The stream of research about how consumers perceive remanufactured products also relates to our research. There are two subcategories of this research. The first subcategory assumes that new and remanufactured products are perceived to be of the same quality by customers. Savaskan et al. [4] examined the optimal collection channel when remanufactured products can be upgraded to the quality of new products. Savaskan and Van Wassenhove [37] further extended their model to a competing retailer setting. Atasu et al. [7] consider a segment of green consumers existing in the market who view the new and remanufactured products to be the same. Another subcategory assumes that consumers have different valuation for new and remanufactured products [12,38,39,40] For instance, Atasu et al. [7] stated that ordinary consumers would value remanufactured products lower. Ma et al. [41] examined the coexistence of new and remanufactured products under two different trade-in programs. Ma et al. [42] investigated original equipment manufacturers’ (OEMs’) remanufacturing license strategy when an independent remanufacturer exists in the market to compete with them. Some researchers try to identify the reason why consumers value new and remanufactured products differently. There are both functional and psychological explanations. The brand equity linked to the original manufacturer [43] and quality uncertainty about the remanufactured products [44] are functional explanations, whereas unpleasant reactions related to contamination [11] and lack of green concerns [45] are psychological explanations. As such, consumers may be reluctant to buy remanufactured products. In this paper, we consider the scenario that consumers can distinguish between new and remanufactured products but feel uncertain about the quality of remanufactured products. Furthermore, consumers’ uncertainty can reduce when more consumers purchase the product. 

Lastly, our research relates to the coexistence of new and remanufactured products (see [46] for a comprehensive review). A basic problem faced by many original equipment manufacturers (OEMs) is whether they should implement remanufacturing, because the existence of remanufactured product can erode the market share of new product [47]. A common suggestion for OEMs is that they should offer remanufactured products only when some specific conditions are satisfied ([9,48]). Ferrer and Swaminathan [49] found that OEMs may decrease the sales prices of new products to increase used products available for remanufacturing when remanufacturing is very profitable. Atasu et al. [7] conclude that, whether a firm can benefit from remanufacturing depends on the existence of green consumers, competition in the OEM market, and product life cycle. Wu and Zhou [12] examined third-party entrances on OEM’s profits and found that an OEM can benefit from the entry when functionality-oriented consumers exist in the market. Yan et al. [40] study the optimal price for new and remanufactured products when consumers’ demand is random. Zhang and He [50] modeled a market choice from the consumers’ perspective when new and remanufactured products coexist in the market. Wu et al. [51] investigated how the assimilation and contrast effect affect firms’ remanufacturing and pricing strategy when facing different remanufacturing scenarios. In this paper, we first consider a firm selling a remanufactured product as a base setting. We then extend our model to a competitive setting, with new and remanufactured products coexisting in the same market and examine how the competition between new and remanufactured product affects firms’ pricing and the government’s subsidy strategy.

## 3. Model Setting

The supply chain for the TOR program which is implemented in China has three important entities: (1) government offering a unit subsidy to consumers when consumers trade their old product for a remanufactured one; (2) the OEM producing and selling products in the market; and (3) consumers holding old product purchase the remanufactured product when receiving a subsidy. We consider a Stackelberg game between the government and the OEM where the government is the leader and the OEM is the follower. The government first decides the subsidy levels offered to consumers. Then, after observing the government’s subsidy level, the OEM sets the sales price and decides production quantities to maximize its profit. In this section, we detail our description about the government, the firm, and the consumers.

### 3.1. Government

We consider two scenarios for the government: with and without a budget constraint. When the government does not have a budget constraint, the problem is an unbinding problem. When the government has a budget constraint, the government uses a planned budget, denoted as K, to maximize the sales quantity of remanufactured products in a specified period. For instance, the Chinese government stated that “the subsidy period for promoting remanufactured products should be no more than five years”. Based on whether the government provides a subsidy, we develop a two-period model, and a subsidy only exists in the first period. 

Two common objectives for the government are to maximize the welfare of the system or to minimize expenditures. Here, we assume that the government’s goal is to maximize sales quantity of remanufactured products. Such assumption is equivalently to minimize the expenditures and consistent with real TOR practice when the government has a budget constraint and only provides a subsidy in one period. 

The government provides a unit subsidy, s, to consumers, when they participate in the TOR program. Let $D_{1r},D_{2r}$ denote the demand for remanufactured products in the first and second periods. The government’s total subsidy cost is equal to $s.D_{1r}$. Then, we can formulate the government’s optimization problem subject to a given budget:

(1) $$\max\;D_{1r}+D_{2r}$$

(2) $$\mathrm{Subject}\;\mathrm{to}\;s.D_{1r}\leq K$$

### 3.2. OEM

Despite the government’s goal, which is to maximize the sales quantity of remanufactured products, we found that the government would select different types of firms as pilot firms to implement the TOR program. For instance, among the selected automakers or engine manufacturers, Sinotruk Jinan Fuqiang and Weichai focus only on producing remanufactured products, whereas FAW-Volkswagen produces new and remanufactured engines at the same time. We analyze two supply chain settings based on different scenarios. In the first scenario, the OEM only produces and sells remanufactured products in the market. In the second scenario, the OEM produces new and remanufactured products, and sells the two products at the same market. We refer to the two settings as Setting 1 and Setting 2 in this paper. The purpose for analyzing these two scenarios is to investigate how the competition between new and remanufactured products affects firms’ optimal pricing strategy and the government’s subsidy policy. 

In a two-period model, the OEM seeks to maximize its profits over the two periods. In Setting 1, when the OEM offers only the remanufactured products, the firm sets the selling price of remanufactured products, $p_{1r}$, in period 1. In period 2, the firm determines the selling price, $p_{2r}$, after observing consumers’ remanufactured products demand in period 1. Consumers’ demand in the two periods is denoted as $D_{1r}$ and $D_{2r}$, respectively. In Setting 2, besides determining the selling price of remanufactured products, the firm has to set the selling price of new products, $p_{1n}$ and $p_{2n}$, in the two periods when the OEM also offers the new products. Consumers’ demand for new products over the two periods can be denoted as $D_{1n}$ and $D_{2n}$, respectively. For ease of exposition, we assume that the firm’s discount factor in the two periods is 1. We normalize the production cost for remanufactured products to 0, and therefore the production cost for new products is c, to denote cost saving from remanufacturing.

### 3.3. Consumers

Network externality only occurs in the second period because consumers’ utility increases as more consumers purchase in the first period. This effect can apply to both new and remanufactured products because consumers’ decision of buying either a new or remanufactured product is, to some extent, influenced by other consumers’ purchasing behavior. However, compared to new products, consumers feel more uncertain about the quality of remanufactured products, which makes them more reluctant or even not make a purchase. Hence, in order to focus on remanufacturing industry development, we assume that network externality only occurs among consumers who buy the remanufactured products. A consumer’s utility from buying a remanufactured product would increase by $\lambda D_{1r}$, where the parameter λ∈(0,1) represents the strength of network externality. 

Consumers with heterogeneous valuation are uniformly distributed on the interval [0,1]. Without loss of generality, the market size is normalized to 1. We assume that consumers’ willingness to pay for the remanufactured product is less than that for the new product. Consumers’ willingness to pay for a new product is θ∈[0,1], whereas consumers’ willingness to pay for a remanufactured product is αθ, where 0<α<1. The quality and quantity of the used products are guaranteed in the TOR program, which can improve the quality of the remanufactured products in the second period. As such, the quality of the firms’ remanufactured products in the second period is improved to βθ, where 0<α<β<1. All consumers are strategic and determine their purchase time to maximize their expected utility. Let δ∈[0,1] denote consumers’ discount factor; a higher value means that consumers are more strategic so that the second period utility weighs more heavily when they make purchase decisions in the first period. We assume α,β and δ satisfy the following conditions: 0<βδ<δ<α<β<1. 

A consumer will have a net utility of $u_{1n}=\theta -p_{1n}$ from purchasing a new product in the first period, and a net utility of $u_{2n}=\delta (\theta -p_{2n})$ from purchasing a new product in the second period. Similarly, the net utility of purchasing a remanufactured product in the first period is $u_{1r}=\alpha \theta -p_{1r}+s$, and the net utility of purchasing a remanufactured product in the second period is $u_{2r}=\delta (\beta \theta -p_{2r}+\lambda D_{1r})$. 

Consumers will compare these utilities and make a purchase if, and only if, the net utility is positive. We assume that all strategic consumers follow a “threshold purchasing policy” in equilibrium [36], to avoid repetition. We do not consider the special case of all consumers purchasing in the same period. Hence, without loss of generality, we consider the most general case in which consumers with higher valuation will purchase in the first period, whereas consumers with lower valuation will purchase in the second period. Therefore, consumers’ threshold purchasing policy when only remanufactured products exist in the market can be described as follows: (a) Consumers with valuation $\theta \in \left[\tau_{1r},1\right]$ will purchase remanufactured products in the first period, and (b) consumers with valuation $\theta \in \left[\tau_{2r},\tau_{1r}\right]$ will purchase remanufactured products in the second period. Consumers’ threshold purchasing policy when new and remanufactured products coexist in the market can be described as follows: (a) Consumers with valuation $\theta \in \left[\tau_{1n},1\right]$ will purchase new products in the first period; (b) consumers with valuation $\theta \in \left[\tau_{1r},\tau_{1n}\right]$ will purchase remanufactured products in the first period; (c) consumers with valuation $\theta \in \left[\tau_{2n},\tau_{1r}\right]$ will purchase new products in the second period; and (d) consumers with valuation $\theta \in \left[\tau_{2r},\tau_{2n}\right]$ will purchase remanufactured products in the second period. Note that the thresholds $\tau_{2r},\tau_{2n},\tau_{1r}$, and $\tau_{1n}$ depend on $p_{2r},p_{2n},p_{1r},p_{1n},\alpha ,\beta ,\lambda$, and δ. 

Consumers’ demands for new and remanufactured products can be easily obtained when consumers follow the above threshold purchasing policy, i.e., $D_{1n}=1-\tau_{1n}$, $D_{1r}=\tau_{1n}-\tau_{1r}$, $D_{2n}=\tau_{1r}-\tau_{2n}$, and $D_{2r}=\tau_{2n}-\tau_{2r}$ when new and remanufactured products coexist in the market. According to the Karush–Kuhn–Tucker’s necessary conditions, some of the demands may be empty or do not exist. However, we only consider the scenario when all the demands are positive and the condition of $\tau_{2r}<\tau_{2n}<\tau_{1r}<\tau_{1n}$ is satisfied. 

## 4. Model Analysis

To investigate the implications of a government subsidy and externality on the equilibrium outcomes, we analyze two supply chain settings. We first analyze Setting 1 in Section 4.1, where the firm only produces remanufactured products. In Section 4.2, we then analyze Setting 2, where new and remanufactured products are offered to the same market. We consider two scenarios for the two settings: The government has or does not have a budget constraint. Let $\prod^{i}$ denote the OEM’s profit in model j, where $j\in \left\{S1_{N},S1_{B},S2_{N},S2_{B}\right\}$ index Setting 1 without and with a budget constraint, and Setting 2 without and with a budget constraint. We use backward induction to solve the problem. 

### 4.1. Setting 1: Only Remanufactured Products Exist in the Market

When only remanufactured products exist in the market, we first analyze the scenario when the government does not have a budget constraint, and then analyze the scenario when the government has a budget constraint.

#### 4.1.1. The Government Does Not Have a Budget Constraint

We begin our analysis by analyzing consumers’ purchasing behavior in the second period, since consumers are strategic. Note that consumers with valuation $\theta \in \left[\tau_{1r},1\right]$ will purchase remanufactured products in the first period, and the demand in this period is $D_{1r}=1-\tau_{1r}$. A consumer remaining in the second period will purchase the remanufactured product if, and only if, $u_{2r}=\delta (\beta \theta -p_{2r}+\lambda D_{1r})>0\Rightarrow \tau_{2r}=\frac{p_{2r}-\lambda D_{1r}}{\beta }$. In this case, the demand in the second period is $D_{2r}=\tau_{1r}-\tau_{2r}=\tau_{1r}-\frac{p_{2r}-\lambda (1-\tau_{1r})}{\beta }$. The OEM maximizes its profit in the second period by the setting price, $p_{2r}$, as follows:

(3) $$\prod_{2}^{B}=\underset{p_{2r}}{\max\;}p_{2r}.D_{2r}(p_{2r})=\underset{p_{2r}}{\max\;}p_{2r}.\left(\tau_{1r}-\frac{p_{2r}-\lambda (1-\tau_{1r})}{\beta }\right)$$

We obtain the optimal price, demand, and profit in the second period by using first-order condition with respect to $p_{2r}$.

(4) $${p_{2r}}^{*}(\tau_{1r})=\frac{\tau_{1r}(\beta -\lambda )+\lambda }{2};{D_{2r}}^{*}(\tau_{1r})=\frac{\tau_{1r}(\beta -\lambda )+\lambda }{2\beta };\prod_{2}^{B*}(\tau_{1r})=\frac{{\left(\tau_{1r}(\beta -\lambda )+\lambda \right)}^{2}}{4\beta }$$

Next, we investigate the OEM’s problem in the first period, where the OEM maximizes its total profits over the two periods by the setting price, $p_{1r}$. In the first period, a consumer will purchase a remanufactured product if, and only if, his or her net utility from buying is non-negative and is higher than that from purchasing in the second period, i.e., $\alpha \theta -p_{1r}+s>0$ and $\alpha \theta -p_{1r}+s>\delta (\beta \theta -p_{2r}+\lambda D_{1r})$. By rational expectation equilibrium, a consumer with valuation $\theta \in \left[\tau_{1r},1\right]$ will buy the remanufactured products in the first period, so that $\alpha \tau_{1r}-p_{1r}+s=\delta (\beta \tau_{1r}-p_{2r}+\lambda D_{1r})$. By substituting ${p_{2r}}^{*}(\tau_{1r})=\frac{\tau_{1r}(\beta -\lambda )+\lambda }{2}$, the equilibrium price $p_{1r}$ can be expressed as follows:

(5) $$p_{1r}(\tau_{1r})=\frac{2s+2\tau_{1r}\alpha -\tau_{1r}\beta \delta -(1-\tau_{1r})\lambda \delta }{2}$$

Hence, the OEM’s optimal problem in the first period can be formulated as follows:

(6) $$\prod^{S1*}=\underset{p_{1r}}{\max\;}p_{1r}.D_{1r}+\prod_{2}^{B*}(\tau_{1r})$$

By substituting $p_{1r}(\tau_{1r})$ and $\prod_{2}^{B*}(\tau_{1r})$, the OEM’s problem can be reformulated to the following:

(7) $$\prod^{S1*}=\underset{\tau_{1r}\leq 1}{\max\;}\left\{\frac{2s+2\tau_{1r}\alpha -\tau_{1r}\beta \delta -(1-\tau_{1r})\lambda \delta }{2}.(1-\tau_{1r})+\frac{{\left(\tau_{1r}(\beta -\lambda )+\lambda \right)}^{2}}{4\beta }\right\}$$

To ensure the objective function is concave in $\tau_{1r}$, we impose the condition of $\alpha >\frac{\left(\beta -\lambda )(\beta +2\beta \delta -\lambda \right)}{4\beta }$. The optimal value of $\tau_{1r}$ can be obtained from the first-order conditions, which can in turn be used to solve the equilibrium outcomes for the OEM in both periods. We summarize the optimal results in the following proposition.

**Proposition** **1.** *In Setting 1, the OEM only produces the remanufactured products, and there is no budget constraint. The optimal prices, demands, and total profit can be expressed as follows:*$p_{1r}^{S1_{N}*}=\frac{s+2\alpha }{2}+\frac{-4\alpha^{2}\beta +2\alpha \beta (\beta -(1+\delta )\lambda )+(\beta -\lambda )(\beta^{2}\delta^{2}-s(\beta -\lambda ))}{8\alpha \beta -2(\beta -\lambda )(\beta +2\beta \delta -\lambda )}$, $p_{2r}^{S1_{N}*}=\frac{2\alpha \beta (\beta +\lambda )-(2s+\beta \delta )\beta (\beta -\lambda )}{8\alpha \beta -2(\beta -\lambda )(\beta +2\beta \delta -\lambda )}$, $D_{1r}^{S1_{N}*}=\frac{\beta (2s+2\alpha -\beta -\beta \delta +\lambda )}{4\alpha \beta -(\beta -\lambda )(\beta +2\beta \delta -\lambda )}$, $D_{2r}^{S1_{N}*}=\frac{2\alpha (\beta +\lambda )-(2s+\beta \delta )(\beta -\lambda )}{8\alpha \beta -2(\beta -\lambda )(\beta +2\beta \delta -\lambda )}$, $\prod^{S1_{N}*}=\frac{4s^{2}\beta +4\alpha \lambda \beta +{\left(2\alpha -\beta \delta \right)}^{2}\beta +4s(2\alpha -\beta (1+\delta )+\lambda )\beta }{16\alpha \beta -4(\beta -\lambda )(\beta +2\beta \delta -\lambda )}$.

Proposition 1 illustrates that the OEM’s optimal prices, demands, and profit are closely related to consumers’ valuation for remanufactured products, before and after the subsidy (α,β), consumers’ strategic behavior (δ), network externality (λ), and government subsidy (s). For instance, consumers’ demand in the first period increases with the government subsidy, whereas it decreases with the government subsidy in the second period, though the government only provides the subsidy in the first period. This indicates that more consumers would make a purchase in the first period rather than delay and wait until more consumers purchase. We first analyze the structural properties when the government does not have a budget constraint, and then analyze the scenario when the government has a budget constraint. 

The structural properties of the optimal results without a budget constraint, under Setting 1, are summarized in the following corollary.

**Corollary** **1.** *The optimal results without a budget constraint, when only remanufactured products exist in the market have the following characteristics:*

*(a) The OEM implements a markup pricing* ($p_{1r}^{S1_{N}*}<p_{2r}^{S1_{N}*}$) *when* $0<s<s_{1}$, *and implements a markdown pricing* ($p_{1r}^{S1_{N}*}>p_{2r}^{S1_{N}*}$) *when* $s_{1}<s<1$, *where* $s_{1}=\frac{\beta (2\alpha -\beta \delta +\delta \lambda )(\beta +\beta \delta -2\alpha )+2\alpha \lambda^{2}}{4\alpha \beta -2(\beta -\lambda )(\beta \delta -\lambda )}$;

*(b) The optimal demands are so that*$D_{1r}^{S1_{N}*}<D_{2r}^{S1_{N}*}$when$0<s<s_{2}$, *and*$D_{1r}^{S1_{N}*}>D_{2r}^{S1_{N}*}$when$s_{2}<s<1$, *where*$s_{2}=\frac{\beta^{2}(2+\delta )-\beta \lambda (2-\delta )-2\alpha (\beta -\lambda )}{6\beta -2\lambda }$;

*(c) The OEM’s profit,*$\prod^{S1_{N}*}$, *is always increasing with the government subsidy level,*s.

When the government subsidy level, s, is low, the markup pricing strategy is an optimal strategy for the OEM, i.e., $p_{1r}^{S1_{N}*}<p_{2r}^{S1_{N}*}$. A low price $p_{1r}^{S1_{N}*}$ will cause more consumers to make the purchase in the first period, which would reduce remaining consumers’ uncertainty level about the quality of the remanufactured products in the second period. As a result, remaining consumers in the second period would have a higher valuation for the product, and hence, the OEM can charge a higher price in the second period. It is also interesting to note that many consumers would wait and delay their purchase when the government subsidy is low, even though the OEM offers a lower price in the first period. Consumers’ uncertainty about the quality of the remanufactured products discourages them to purchase in the first period. Consumers’ uncertainty and network externality can improve the OEM’s profit because it allows the firm to charge a higher price to the majority of consumers remaining in the market in the second period. 

When the government subsidy level, s, is high, markdown pricing strategy is optimal for the OEM, i.e., $p_{1r}^{S1_{N}*}>p_{2r}^{S1_{N}*}$. Many consumers who formerly want to wait would purchase the product in the first period, when the government subsidy level is high. Consumers would have a higher utility for the product because of the subsidy, and therefore, the OEM can extract higher surplus from these consumers by charging a higher price. Fewer consumers delay their purchase to the second period, though the firm increases the prices in the first period. The higher subsidy can greatly improve consumers’ utility and ease their uncertainty about the quality of the remanufactured products. 

Finally, the OEM’s profit increases with the government subsidy. The OEM can adjust its pricing strategy with respect to different levels of government subsidy, which influence the firm’s ability to extract surplus from consumers. In the presence of externality, consumers’ purchase decision directly affects consumers’ decision in the second period. The OEM can exploit this by implementing markup or markdown pricing strategy. 

**Figure 1:**
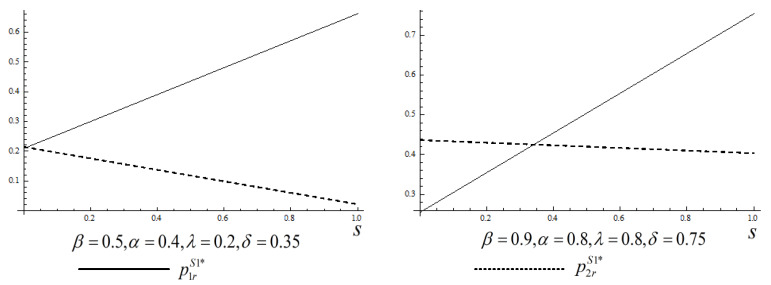
Original equipment manufacturer (OEM)’s pricing over two periods.

From Corollary 1, we know that, given consumers’ valuation for the remanufactured products over the two periods (α and β), consumers’ strategic behavior (δ) and externality effect (λ) have an impact on a firm’s pricing strategy and consumers’ demands over the two periods. However, it is difficult to analyze their monotonicity. Therefore, we use a numerical example to show their impact. The impact of these parameters on a firm’ two periods pricing strategy or consumers’ demands is very similar. Hence, we only investigate their impact on a firm’s pricing. The numerical results show that the impact of consumers’ strategic behavior (δ) and externality effect (λ) on a firm’s two periods’ pricing strategy are not significant. Here, we only present when α and β are large (β=0.9,α=0.8,λ=0.8,δ=0.75), and when α and β are small (β=0.5,α=0.4,λ=0.2,δ=0.35), respectively (Figure 1). 

Figure 1 shows that, when (1) consumers’ valuation for the remanufactured products over the two periods (2) consumers’ strategic behavior and (3) externality effect are high, the OEM’s pricing strategy over the two periods is consistent with Corollary 1. Namely, the OEM should employ a markup pricing strategy when the government subsidy level, s, is small whereas the OEM should employ a markdown pricing strategy when s is large. However, when (1) consumers’ valuation for the remanufactured products over the two periods (2) consumers’ strategic behavior and (3) externality effect are small; therefore, the OEM should always implement a markdown pricing strategy. When consumers’ valuation for the remanufactured products is low, they are more reluctant to make a purchase. As a result, the OEM has to decrease selling price in the second period, to attract more consumers to purchase the remanufactured products. 

#### 4.1.2. The Government Has a Budget Constraint

When the government has a budget constraint, the problem becomes binding. The government acts as the Stackelberg leader in the game, whereas the OEM is the follower. We solve the problem backward. Consumers’ purchase decisions and firms’ optimal results are the same with the results without a budget constraint. Therefore, we only need to consider the government optimal problem with a budget constraint. As stated in the previous section, the government’s optimal problem can be reformulated as follows, by substituting $D_{1r}^{S1_{N}*}$:

(8) $$\max\;D_{1r}^{S1_{N}*}+D_{2r}^{S1_{N}*}$$

(9) $$\mathrm{Subject}\;\mathrm{to}\;s.\left(\frac{\beta (2s+2\alpha -\beta -\beta \delta +\lambda )}{4\alpha \beta -(\beta -\lambda )(\beta +2\beta \delta -\lambda )}\right)\leq K$$

We summarize the optimal results with a budget constraint in the following proposition.

**Proposition** **2.** *When the OEM produces only the remanufactured products, the government has a budget constraint, and the constraint is binding, so the optimal demand is*$D_{1r}^{S1_{B}*}=\frac{\beta (2s^{S1_{B}*}+2\alpha -\beta -\beta \delta +\lambda )}{4\alpha \beta -(\beta -\lambda )(\beta +2\beta \delta -\lambda )}$, *and the optimal subsidy level is*$s^{S1_{B}*}=\frac{-b_{1}+\sqrt{{b_{1}}^{2}-4a_{1}c_{1}}}{2a_{1}}$, *where*$a_{1}=2\beta$, $b_{1}=\beta (2\alpha -\beta -\beta \delta +\lambda )$, *and*$c_{1}=-K\left(4\alpha \beta -(\beta -\lambda )(\beta +2\beta \delta -\lambda )\right)$.

The firm’s optimal prices, consumers’ demands, and the firms’ total profit can be obtained by substituting the optimal subsidy level, $s^{S1_{B}*}$. Proposition 2 suggests that the budget constraint is binding. The optimal subsidy level is increasing with the constraint, which indicates that the government can still increase consumers’ demand for remanufactured product with a limited budget. We analyze the structural properties in the following corollary.

**Corollary** **2.** *The optimal results with a budget constraint, when only remanufactured products exist in the market, have the following characteristics:*

*(a) The OEM always implements a markup pricing* ($p_{1r}^{S1_{B}*}<p_{2r}^{S1_{B}*}$) *when* $s^{S1_{B}*}<s_{1}$, *and it always implements a markdown pricing* ($p_{1r}^{S1_{B}*}>p_{2r}^{S1_{B}*}$) *when* $s_{1}<s^{S1_{B}*}$;

*(b) The optimal demands are so that*$D_{1r}^{S1_{B}*}<D_{2r}^{S1_{B}*}$when$s^{S1_{B}*}<s_{2}$, *and*$D_{1r}^{S1_{B}*}>D_{2r}^{S1_{B}*}$when$s_{2}<s^{S1_{B}*}$;

*(c) The OEM’s profit,*$\prod^{S1_{B}*}$, *is always increasing with the government subsidy level,*$s^{S1_{B}*}$.

Corollary 2 implies similar implications with Corollary 1. We note that there are several distinctions of the firm’s strategy when the government is with and without a budget constraint. The OEM may have to change from a markup pricing strategy to a markdown pricing strategy when the government increases its subsidy level or when there is no budget constraint. However, there is no pricing strategy change for the OEM when there is a budget constraint for the government. The OEM should always employ a markup pricing strategy when the government subsidy is low, whereas the OEM should always implement a markdown strategy when the government subsidy is high. The same applies to consumers’ demands for remanufactured products. The OEM’s total profit still increases with the government subsidy level, although there is an upper limit. 

### 4.2. Setting 2: New and Remanufactured Products Coexist in the Market

When new and remanufactured products are offered to the market, we first analyze the scenario when the government does not have a budget constraint, and then analyze the scenario when the government has a budget constraint.

#### 4.2.1. The Government Does Not Have a Budget Constraint

In this section, we investigate the equilibrium results when new and remanufactured products coexist in the market. In this setting, consumers who previously leave the market because of the uncertainty about the quality of the remanufactured products can now purchase the new products instead. Network externality occurs among consumers who purchase the remanufactured products. For the government, subsidies can encourage more consumers to purchase remanufactured products in the first period, but it can also lead to more consumers delaying their purchase decisions, in order to observe other consumers’ purchase decision, to lower their uncertainty. For the selected OEM, it should take the possible cannibalization problem into consideration, because the existence of remanufactured products may erode the market share of its new products. For consumers, in addition to anticipating the firm’s pricing strategy, they would also consider whether other consumers have purchased the remanufactured products in the first period or not. Using the similar methods of the previous section, we can obtain the following optimal results (see Appendix A for a detailed analysis).

**Proposition** **3.** *In Setting 2, the OEM produces the new and remanufactured products, and there is no budget constraint. The optimal prices, demands, and total profit can be expressed as follows:*$p_{1n}^{S2_{N}*}=\frac{\left(\begin{array}{l} A(1+s-5\alpha +2(1+\alpha )\delta -\delta^{2}+c(1-2\alpha +\delta )(1+\delta ))\\ +2c(1-\alpha )\beta \lambda +\lambda^{2}{\left(2-\delta \right)}^{2}+c\lambda^{2}(2-\delta^{2}) \end{array}\right)}{2(AB+C)}$, $p_{1r}^{S2_{N}*}=s+\frac{\left(\begin{array}{l} (2\alpha -\delta )\left(A(2s-2\alpha +\delta )+(2-\delta )\lambda^{2}+4(1-\alpha )\beta \lambda \right)\\ +c\left(A(2\alpha (1-\delta )+\delta^{2})\right)+2\alpha \lambda^{2}(1-\delta )+\lambda^{2}(2-\delta )\delta \end{array}\right)}{2(AB+C)}$, $p_{2n}^{S2_{N}*}=\frac{c}{2}+\frac{\left(A(c+2s-2\alpha +\delta +c\delta )+4c(1-\alpha )\beta \lambda +(2+c(1-\delta )-\delta )\lambda^{2}\right)}{2(AB+C)}$, $p_{2r}^{S2_{N}*}=\frac{\beta \left(\begin{array}{l} A(c+2s-2\alpha +\delta +c\delta )-2\lambda (1-\beta )(\alpha +s(3-2\delta )-1)\\ -2c\lambda (1-3\beta +2\alpha (1-\delta +\beta \delta ))+(1-c)(2-\delta )\lambda^{2} \end{array}\right)}{2(AB+C)}$, $D_{1n}^{S2_{N}*}=1-\frac{\left(2B(1+c+s-\alpha )(1-\beta )\beta +c\beta \lambda B+\lambda^{2}(2+c-(1+c)\delta )\right)}{\left(AB+C\right)}$, $D_{1r}^{S2_{N}*}=\frac{\left(2(1-c(1+2\alpha (1-\delta ))-\alpha )(1-\beta )\beta +(s-c\beta \lambda )(3-2\delta )\right)}{\left(AB+C\right)}$, $D_{2n}^{S2_{N}*}=\frac{\left(\begin{array}{l} 4(1-\alpha )\beta (2s(1-\beta )-c\beta +2\alpha (2c+\beta -1)-(c+\beta +c\beta -1)\delta )\\ +2\beta (3c+3s-1+\alpha -2(s+c\alpha )\delta )\lambda +(1-c)(2-\delta )\lambda^{2} \end{array}\right)}{2(AB+C)}$, $D_{2r}^{S2_{N}*}=\frac{\left(2c(1-\alpha )\beta B+(1-c-3s-\alpha -2c\alpha +2(s+c\alpha )\delta )\lambda \right)}{\left(AB+C\right)}$, $\prod^{S2_{N}*}=(p_{1n}^{S2_{N}*}-c)D_{1n}^{S2_{N}*}+p_{1r}^{S2_{N}*}D_{1r}^{S2_{N}*}+(p_{2n}^{S2_{N}*}-c)D_{2n}^{S2_{N}*}+p_{2r}^{S2_{N}*}D_{2r}^{S2_{N}*}$, *where*A=4(1−α)(1−β)β, B=1−4α+2δ*and*$C=(3-2\delta )\lambda^{2}$*are used for simplifying the mathematical derivations.*

Like Proposition 1, Proposition 2 shows that the OEM’s optimal price and consumers’ demands depend on the consumers’ valuation for the remanufactured product, consumers’ strategic behavior, network externality, and cost saving from remanufacturing. The government subsidy not only affects the firm’s price and consumers’ demands for remanufactured products but also affects the firms’ price and consumers’ demands for new products. In the following, we analyze the structural properties of the equilibrium results when new and remanufactured products coexist in the market and there is no budget constraint for the government.

**Corollary** **3.** *The optimal results without a budget constraint, when new and remanufactured products coexist in the market, have the following characteristics:*

*(a) The OEM implements a markup pricing* ($p_{1n}^{S2_{N}*}<p_{2n}^{S2_{N}*}$) *for new products when* $s_{3}<s<1$, *and it implements a markdown pricing* ($p_{1n}^{S2_{N}*}>p_{2n}^{S2_{N}*}$) *for new products when* $0<s<s_{3}$; *the OEM implements a markup pricing* ($p_{1r}^{S2_{N}*}<p_{2r}^{S2_{N}*}$) *for remanufactured products when* $0<s<s_{4}$, *and it implements a markdown pricing* ($p_{1r}^{S2_{N}*}>p_{2r}^{S2_{N}*}$) *for remanufactured products when* $s_{4}<s<1$, *where* $s_{3}=1-c-3\alpha +2c\alpha +\delta -c\delta +2\alpha \delta -2c\alpha \delta -\delta^{2}+c\delta^{2}-\frac{c\lambda }{2(1-\beta )}+\frac{(1-c)(2-\delta )(1-\delta )\lambda^{2}}{B}$, $s_{4}=\frac{\left(\begin{array}{l} A(2\alpha -\delta )(2\alpha -\beta -\delta )+\frac{A\lambda }{2}-(2-\delta )(2\alpha -\beta -\delta )\lambda^{2}+Ac(\beta -2\alpha (1-\delta )+(\beta -\delta )\delta )\\ -2c\beta (1-3\beta -2\delta +2(3-2\alpha +\beta \delta )\alpha )-2\alpha (1-\delta )\lambda^{2}-(2-\delta )(\beta +\delta )\lambda^{2} \end{array}\right)}{2A(1-2\alpha -\beta +\delta )+2\beta (1-\beta )(3-2\delta )\lambda +2C}$;

*(b) The optimal demands for new products are so that*$D_{1n}^{S2_{N}*}<D_{2n}^{S2_{N}*}$when$0<s<s_{5}$, *and*$D_{1n}^{S2_{N}*}>D_{2n}^{S2_{N}*}$when$s_{5}<s<1$; *the optimal demands for remanufactured products are so that*$D_{1r}^{S2_{N}*}<D_{2r}^{S2_{N}*}$when$0<s<s_{6}$, *and*$D_{1r}^{S2_{N}*}>D_{2r}^{S2_{N}*}$when$s_{6}<s<1$, *where*$s_{5}=\frac{\left(\begin{array}{l} A(1-2\alpha +\delta )+4c\beta (4\alpha^{2}-1+2\beta -5\alpha \beta -(1+\alpha +(\alpha -3)\beta )\delta )\\ +2(1-\alpha )\beta (1-2c(2+\delta ))\lambda -(1-c)\delta \lambda^{2} \end{array}\right)}{4(1-\beta )\beta (3-6\alpha +2\delta )+2\beta (3-2\delta )\lambda }$, $s_{6}=\frac{\left(\begin{array}{l} 2\beta (1-\alpha -\beta +\alpha \beta -c(2-\beta +2\delta +(4\alpha -3-2\beta -2(2-\beta )\delta )\alpha ))\\ +\lambda (\alpha -1+c(1-3\beta +2\alpha (1-\delta )+2\beta \delta )) \end{array}\right)}{\left(3-2\delta )(2(1-\beta )\beta -\lambda \right)}$;

*(c) The OEM’s profit*$\prod^{S2_{N}*}$is always increasing with the constraint,K.

Similar to Corollary 1, Corollary 3 shows that the OEM also has to implement markup or markdown pricing strategy according to different subsidy levels. When the government subsidy level is low, markup pricing strategy is optimal for the sale of remanufactured products. When the government subsidy level is high, the OEM should implement a markdown pricing strategy for the remanufactured products. However, the pricing strategy for the new products is completely different. The OEM should employ a markdown pricing strategy for the new products when the subsidy level is low. When the government subsidy level is high, the OEM should employ a markup pricing strategy for the new products. This implies that the firm should offer two different pricing strategies for the new and remanufactured products when both new and remanufactured products are offered to the market. The profit losses from one product can be offset by the other product. Consumers’ demands for new products decrease when the firm increases the sale price for new products, and when the price increases, consumers’ demands decrease. Consumers’ demands for remanufactured products exhibit the similar trends with the sale price of the remanufactured products, because network externality effects exist among consumers who buy remanufactured products. A low price, $p_{1r}^{S2_{N}*}$, will induce more consumers to purchase the remanufactured products in the first period, which can increase consumers’ utility in the second period. As a result, more remaining consumers would still make a purchase, even though the firm increases the sale price for the remanufactured products. Finally, the OEM’s profit increases with the government subsidy because the firm can adjust its pricing strategies according to different subsidy levels, which enables them to extract more surpluses from consumers. 

We use a numerical example to show the impact of consumers’ valuation for the remanufactured products over the two periods (α and β), consumers’ strategic behavior (δ), and externality effect (λ) on firm’s pricing strategy, demands, and profits. However, we find that their trends replicate Figure 1. Hence, we omit presenting the results. 

#### 4.2.2. The Government Has a Budget Constraint

When the OEM produces new and remanufactured products in the market, the government provides a subsidy to consumers when they buy remanufactured products, but with a budget constraint, the problem becomes binding. Using the similar methods of the previous section, we can reformulate the government’s optimal problem as follows:

(10) $$\max\;D_{1r}^{S2_{N}*}+D_{2r}^{S2_{N}*}$$

(11) $$\mathrm{Subject}\;\mathrm{to}\;s.\left(\frac{\left(2(1-c(1+2\alpha (1-\delta ))-\alpha )(1-\beta )\beta +(s-c\beta \lambda )(3-2\delta )\right)}{\left(AB+C\right)}\right)\leq K$$

The optimal results with a budget constraint can be summarized in the following proposition.

**Proposition** **4.** *When new and remanufactured products coexist in the market, the government provides subsidy to consumers, but with a budget constraint, and the constraint is binding, the optimal demand is*$D_{1r}^{S2_{B}*}=\frac{\left(2(1-c(1+2\alpha (1-\delta ))-\alpha )(1-\beta )\beta +(s^{S2_{B}*}-c\beta \lambda )(3-2\delta )\right)}{\left(AB+C\right)}$, *and the optimal subsidy level is*$s^{S2_{B}*}=\frac{-b_{2}+\sqrt{{b_{2}}^{2}-4a_{2}c_{2}}}{2a_{2}}$, *where*$a_{2}=(3-2\delta )$, $b_{2}=2(1-c(1+2\alpha (1-\delta ))-\alpha )(1-\beta )\beta -c\beta \lambda (3-2\delta )$, *and*$c_{1}=-K\left(AB+C\right)$.

We can obtain the firms’ optimal prices, consumers’ demands, and the firm’s total profits by substituting the optimal subsidy level, $s^{S2_{B}*}$. Proposition 4 shows that the optimal subsidy level is increasing with the budget constraint, which implies that consumers’ demands for remanufactured products in the first period increase with the government subsidy, even though new and remanufactured products coexist in the market. We analyze the structural properties of the scenario when the government has a budget constraint and new and remanufactured products coexist in the market in the following corollary.

**Corollary** **4.** *The optimal results with a budget constraint, when new and remanufactured products coexist in the market, have the following characteristics:*

*(a) The OEM always implements a markdown pricing* ($p_{1n}^{S2_{B}*}>p_{2n}^{S2_{B}*}$) *strategy for the new products when* $s^{S2_{B}*}<s_{3}$, *and implements a markup pricing* ($p_{1n}^{S2_{B}*}<p_{2n}^{S2_{B}*}$) *when* $s^{S2_{B}*}>s_{3}$; *for the remanufactured products, the OEM always implements a markup pricing* ($p_{1r}^{S2_{B}*}<p_{2r}^{S2_{B}*}$) *when* $s^{S2_{B}*}<s_{4}$, *and implements markdown pricing* ($p_{1r}^{S2_{B}*}>p_{2r}^{S2_{B}*}$) *when* $s^{S2_{B}*}>s_{4}$;

*(b) The optimal demands for the new products are so that*$D_{1n}^{S2_{B}*}<D_{2n}^{S2_{B}*}$when$s^{S2_{B}*}<s_{5}$, *and*$D_{1n}^{S2_{B}*}>D_{2n}^{S2_{B}*}$when$s^{S2_{B}*}>s_{5}$; *the optimal demands for the remanufactured products are so that*$D_{1r}^{S2_{B}*}<D_{2r}^{S2_{B}*}$when$s^{S2_{B}*}<s_{6}$, *and*$D_{1r}^{S2_{B}*}<D_{2r}^{S2_{B}*}$when$s^{S2_{B}*}>s_{6}$;

*(c) The OEM’s optimal profit,*$\prod^{S2_{B}*}$, *is always increasing with the constraint,*K.

Like Corollary 2, Corollary 4 implies similar implications. When the government has a budget constraint, the firm does not need to adjust its pricing strategy from markup to markdown. The OEM shall always employ markup or markdown pricing strategy in response to the specific government subsidy level. When the pricing strategy is fixed, consumers’ demands in the second period would always be higher or lower than consumers’ demands in the first period. The firm’s total profits still increase with the government subsidy, despite the budget constraint. 

## 5. Discussion on Government Subsidy with a Budget Constraint

In the former section, we investigated the firm’s optimal strategy under two settings (i.e., Setting 1, where an OEM only sells remanufactured goods, and Setting 2, where the OEM sells both remanufactured and new goods), with two scenarios: with and without a government budget constraint. In this section, we examine the impact of a government subsidy on firm’s total sale of new and remanufactured products under the two settings. Consistent with the practice of the TOR program, the analyses focus on the scenario with a budget constraint. We first compare the total sale of remanufactured products in the two settings in Proposition 5. Then, we analyze the total sale of products (new and remanufactured) in the two settings in Proposition 6. Finally, we analyze the firm’s profits under both settings, when there is a budget constraint in Proposition 7.

**Proposition** **5.** *The OEM’s total sale of remanufactured products in Setting 1 is larger than that of Setting 2* ($D_{1r}^{S1_{B}*}+D_{2r}^{S1_{B}*}>D_{1r}^{S2_{B}*}+D_{2r}^{S2_{B}*}$) *when the constraint level,* K, *is lower, and the OEM total sale of remanufactured products in Setting 1 is smaller than that of Setting 2* ($D_{1r}^{S1_{B}*}+D_{2r}^{S1_{B}*}<D_{1r}^{S2_{B}*}+D_{2r}^{S2_{B}*}$) *when the constraint level,* K, *is higher.*

Proposition 5 shows that the total sale of remanufactured products in Setting 1 is higher than that when new and remanufactured products coexist in the market when the budget constraint is low. However, when the budget constraint is higher, the firm can sell more remanufactured products when the firm produces the new products at the same time. This result provides important implications for the government, which wants to increase the sale quantity of remanufactured products. That is, if it has a high budget for the remanufacturing industry, it should subsidize firms who produce new and remanufactured products. Meanwhile, if it has a low budget, it should offer a subsidy to firms that only produce remanufactured products.

**Proposition** **6.** *The OEM’s total sale of products in Setting 1 is always lower than in Setting 2, that is,* $D_{1r}^{S1_{B}*}+D_{2r}^{S1_{B}*}<D_{1n}^{S2_{B}*}+D_{2n}^{S2_{B}*}+D_{1r}^{S2_{B}*}+D_{2r}^{S2_{B}*}$ *always exist.*

Proposition 6 implies that the total sale quantity of the products when new and remanufactured products coexist in the market is always larger than when only remanufactured products exist in the market. This shows that the firm can increase its market share by producing new products when its remanufacturing activities are subsidized by the government. However, when the budget constraint is relatively low, the sale of remanufactured products in Setting 2 is smaller than that in Setting 1. In that scenario, the sale of new products can offset the firm’s market loss of remanufactured products.

**Proposition** **7.** *The OEM’s total profit in Setting 1 is larger than the OEM’s total profit in Setting 2 when the budget constraint,*K, *is higher, while the OEM’s total profit in Setting 1 is smaller than the OEM’s total profit in Setting 2 when the budget constraint,*K, *is lower.*

Proposition 7 shows that the OEM’s total profit in Setting 1 is larger when the budget constraint is higher. When the budget constraint is lower, the firm’s total profit in Setting 1 is smaller. Proposition 7 implies an opposite policy for the government as compared to Proposition 5. When the budget constraint is low, the total sale of remanufactured products in Setting 1 is higher than that in Setting 2, but this does not increase the firm’s total profit. This indicates that the firm that only produces remanufactured products may not have an incentive to engage in remanufacturing, although the government’s subsidy policy would induce more consumers to purchase the remanufactured products. The firm that produces new and remanufactured products at the same time can benefit from the government’s policy, although consumers’ demands for remanufactured products are insufficient. In addition to selling remanufactured products, the firm can also benefit from selling new products.

**Figure 2:**
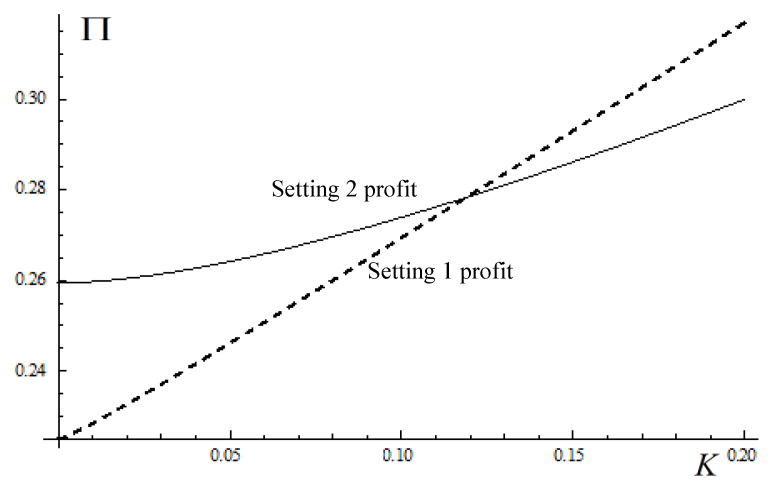
Impact of budget constraint on the OEM’s total profit.

Figure 2 plots the OEM’s total profit in the two settings, with respect to the budget constraint, K, which varies from 0 to 0.2 [29]. We set β=0.9,α=0.6,λ=0.1,δ=0.5,c=0.05. The OEM’s total profit in Setting 1 is depicted by the dotted line. The figure illustrates our results obtained in Proposition 7, and the firm’s total profit in Setting 1 is larger than that of in Setting 2, when the budget constraint is higher. 

## 6. Managerial Implications

In this section, we discuss several implications from the preceding analyses. First, the OEM’s total profit always increases with the government subsidy level, even when there is a budget constraint. Therefore, the government can always offer a subsidy to consumers if it wants to increase the sale quantity of remanufactured products and contribute toward developing a circular economy. Many consumers are reluctant to purchase the remanufactured products because of the uncertainty of the quality of the remanufactured products. The government’s subsidy can induce more consumers to make a purchase and ease consumers’ uncertainty about the remanufactured products. More consumers would buy the remanufactured products in the second period when more consumers buy the product in the first period, because network externality effect exists among consumers who buy the remanufactured products, and consumers in the second period would imitate consumers’ behavior in the first period. 

Second, the government can increase the firm’s total profit, but it does not necessarily increase the sale quantity of remanufactured products at the same time. Comparing the two settings, we find that consumers’ total demands for remanufactured products is higher in Setting 1 when the budget constraint is lower, but the firm’s total profit under this scenario is smaller than the firm’s total profits when the firm also offers new products. When the budget constraint is higher, consumers’ demands for the remanufactured products are higher in Setting 2, but the firm’s total profit is lower in this setting. Therefore, for the OEM, it should only produce remanufactured products when the budget constraint is higher. When the budget constraint is lower, it should produce both new and remanufactured products. The reason is that, when the budget constraint is lower, the number of consumers who buy the remanufactured products is small because of their valuation or uncertainty. As such, the firm should produce new products, in order to increase its market and thereby increase its profit. When the budget constraint is higher, the number of consumers who buy the remanufactured products is large, and the firm can earn more profit from only producing remanufactured products. However, there may be some conflicts with the government whose goal is to increase the sale quantity of remanufactured products. The government subsidy can increase the sale quantity for remanufactured products, but it does not necessarily increase the firm’s total profit. Regardless, a balance must be sought regarding the trade-off between the sale of remanufactured products and firms’ profits. Therefore, for the government, it should always subsidize firms in both settings. 

Ma et al. [41] also examined the effect of government subsidy on the development of remanufacturing industry. However, their focus was on the firms’ optimal decisions, not on the government. Yu et al. [28] and Yu et al. [29] addressed the allocation of government subsidy when the government has different goals. By contrast, we do not address the government subsidy’s allocation problem. We examine which firms the government should select as pilot firms to implement a subsidy policy and the impact of a government subsidy on firms’ optimal decisions. The Chinese government’s TOR program practice is consistent with our results. The government subsidy can increase consumers’ demands for the remanufactured products, but when the budget constraint is low, the OEM will be earning less profit from only producing remanufactured products. Therefore, the government has selected 10 different kinds of automakers or engine manufacturers. Some firms, such as Sinotruk Jinan Fuqiang and Weichai, only produce remanufactured products. Others, such as FAW-Volkswagen, produce new and remanufactured products.

## 7. Conclusions

Remanufacturing has played an important role in shifting supply chains from linear to closed loop. However, the development of the remanufacturing industry faces many challenges. For instance, some challenges include rudimentary recycling practiced by informal recycling firms, insufficient access to used products by formal recycling firms, and consumers’ low willingness to pay for remanufactured products. Therefore, in order to promote the remanufacturing industry’s development and increase the sale quantity of remanufactured products, the Chinese government launched a pilot program to subsidize consumers holding an old product, to trade it for a remanufactured product at the selected firms. More consumers would buy the remanufactured products when they received the subsidy, and consumers would also imitate other consumers’ buying behavior. This imitation behavior is also known as the externality effect that can occur among consumers who buy the remanufactured products. We incorporated the behavior into our model. Besides selecting firms which only produce remanufactured products, the government also selects firms that produce new and remanufactured products. There are two scenarios for the OEM, namely with a budget constraint and without a budget constraint. This paper investigated the optimal outcomes for the government and the OEM, and the conditions under which the OEM should employ a markup or markdown pricing strategy.

From our analyses, we found that, when the OEM only produces remanufactured products, the OEM should change from a markup to a markdown pricing strategy when the subsidy level increases from low to high. The same pricing strategy applies when the government has a budget constraint, but the firm does not need to change its pricing strategy. The optimal subsidy is given when there is a budget constraint. Therefore, the OEM should always maintain a markup or a markdown pricing strategy when the government has a budget constraint. 

When the OEM offers new and remanufactured products in the same market, the OEM should adjust its pricing strategy for new and remanufactured products, according to different subsidy levels. However, the pricing strategy for the new and remanufactured products is completely different. The OEM should implement a markup pricing strategy for the new products if the pricing strategy for the remanufactured products is markdown. 

Through further analysis of the government constraint, we found that the impact of government constraint on consumers’ demands for remanufactured products and firm’s total profit are in opposition to each other. When the government constraint is low, consumers’ demands for remanufactured products in Setting 1 is larger than that in Setting 2, while firm’s total profit in Setting 1 is smaller than that in Setting 2. 

The government can play an important role for the circular or sustainable transition of the economy by focusing on the sustainable development of supply chain through offering subsidization on the remanufacturing industry [41]. This paper provides valuable insights for the government who wants to develop the remanufacturing industry and the OEM which engages in remanufacturing. Our results can provide effective guidelines for OEM to adjust its pricing strategy for new and remanufactured products, according to different subsidy levels, and for the government to set appropriate subsidy levels, to boost the remanufacturing industry [8,31].

Our study contributes to the theory of development by incorporating network externality behavior and consumers’ uncertainty behavior, to examine the impact of government subsidies on the sales of remanufactured products. By accounting for these behaviors, our study provides a clearer, more nuanced explanation of the relationship between subsidies and the sales of remanufactured products. This yields important information for the government and manufacturers to adjust their policy and production to maximize welfare and profits, respectively, under different contexts. Furthermore, the findings are consistent with the actual phenomenon, whereby certain firms only produce remanufactured products, whereas others produce both remanufactured and new products, based on their conditions. This validates the importance of accounting for both network externality behavior and consumers’ uncertainty behavior in the study of government subsidies and the development of the remanufacturing industry. 

There are a few limitations in this study. First, it has only considered active subsidization approach but not passive interventions, such as tax incentives. Therefore, future studies can compare the effectiveness of both types of approaches. Another limitation of this study is that it has not considered promoting the remanufacturing industry from the legal perspective. Hence, in addition to considering economic incentives, future research can examine the impact of legal policies on promoting the remanufacturing industry. Besides, we did not consider the cost structure and competition in the market. It would be very interesting to consider these into future analyses. 

## Appendix A

**Proof of Proposition** **3.** Using backward induction, we first solve a firm’s optimal decision in the second period, and then solve firm’s optimal decision in the first period. In the second period, consumers’ demands for new and remanufactured products $D_{1n}$ and $D_{1r}$ in the first period are realized. Hence, $\tau_{1n}$ and $\tau_{1r}$ are treated as a constant. Consumers’ utility from buying remanufactured products in the second period is influenced by the number of consumers who buy the remanufactured products in the first period ($D_{1r}$), and consumers’ second period utility is increased to $\lambda D_{1r}=\lambda (\tau_{1n}-\tau_{1r})$. Therefore, in the second period, the utility of consumers buy the remanufactured products is $u_{2r}=\delta (\beta \theta -p_{2r}+\lambda (\tau_{1n}-\tau_{1r}))$, and the utility of consumers buy the new products is $u_{2n}=\delta (\theta -p_{2n})$. Recall that the threshold purchasing policy under which a consumer with valuation of θ will buy the remanufactured product if $\theta \in \left[\tau_{2r},\tau_{2n}\right]$, and buy the new product if $\theta \in \left[\tau_{2n},\tau_{1r}\right]$. Using these observations and consumers’ utility functions, thresholds $\tau_{2r}$ and $\tau_{2n}$ simultaneously satisfy the following equations:

(A1) $$\tau_{2r}\beta -p_{2r}+\lambda (\tau_{1n}-\tau_{1r})=0$$

(A2) $$\tau_{2n}\beta -p_{2r}+\lambda (\tau_{1n}-\tau_{1r})=\tau_{2n}-p_{2n}$$

By solving these two equations, we get $\tau_{2r}=\frac{p_{2r}-\lambda (\tau_{1n}-\tau_{1r})}{\beta }$ and $\tau_{2n}=\frac{p_{2n}-p_{2r}+\lambda (\tau_{1n}-\tau_{1r})}{1-\beta }$. In this case, consumers’ demands for the new and remanufactured products in the second period are $D_{2r}=\frac{p_{2n}-p_{2r}+\lambda (\tau_{1n}-\tau_{1r})}{1-\beta }-\frac{p_{2r}-\lambda (\tau_{1n}-\tau_{1r})}{\beta }$, $D_{2n}=\tau_{1r}-\frac{p_{2n}-p_{2r}+\lambda (\tau_{1n}-\tau_{1r})}{1-\beta }$. The OEM maximizes its second period profit by setting price $p_{2n},p_{2r}$, and we have the following:
$\begin{array}{l} \prod_{2}^{S2}=\underset{p_{2n},p_{2r}}{\max\;}\left((p_{2n}-c).D_{2n}+p_{2r}.D_{2r}\right)\\ =\underset{p_{2n},p_{2r}}{\max\;}\left\{(p_{2n}-c)\left(\tau_{1r}-\frac{p_{2n}-p_{2r}+\lambda (\tau_{1n}-\tau_{1r})}{1-\beta }\right)+p_{2r}\left(\frac{p_{2n}-p_{2r}+\lambda (\tau_{1n}-\tau_{1r})}{1-\beta }-\frac{p_{2r}-\lambda (\tau_{1n}-\tau_{1r})}{\beta }\right)\right\} \end{array}$
We obtain the optimal price and profit in the second period by using first-order condition with respect to $p_{2n},p_{2r}$.

(A3) $${p_{2n}}^{*}(\tau_{1r})=\frac{\tau_{1r}+c}{2};{p_{2r}}^{*}(\tau_{1n},\tau_{1r})=\frac{\tau_{1r}(\beta -\lambda )+\tau_{1n}\lambda }{2};$$

(A4) $$\prod_{2}^{S2*}(\tau_{1n},\tau_{1r})=\frac{c^{2}\beta +{\tau_{1r}}^{2}\beta (1-\beta )+{\left(\tau_{1n}-\tau_{1r}\right)}^{2}\lambda^{2}+2c\beta \tau_{1n}\lambda +2c\beta \tau_{1r}(1-\beta +\lambda )}{4\beta (1-\beta )}$$

We next investigate the OEM’s objective function in the first period, where the OEM maximizes its total profits over the two periods by setting price $p_{1n},p_{1r}$. In the first period, consumers will take their second period utility into consideration when making a purchase decision in this period. Recall that the threshold purchasing policy under which a consumer with valuation of θ will buy the remanufactured product if $\theta \in \left[\tau_{1r},\tau_{1n}\right]$, and buy the new product if $\theta \in \left[\tau_{1n},1\right]$. Using the observations and rational expectation equilibrium, thresholds $\tau_{1r}$ and $\tau_{1n}$ simultaneously satisfy the following equations:

(A5) $$\alpha \tau_{1r}-p_{1r}+s=\delta (\tau_{1r}-p_{2n})$$

(A6) $$\alpha \tau_{1n}-p_{1r}+s=\tau_{1n}-p_{1n}$$

By solving these two equations, we get $\tau_{1r}=\frac{p_{1r}-s-\delta p_{2n}}{\alpha -\delta }$ and $\tau_{1n}=\frac{p_{1n}-p_{1r}+s}{1-\alpha }$. Substituting ${p_{2n}}^{*}(\tau_{1r})=\frac{\tau_{1r}+c}{2}$, the equilibrium price $p_{1n},p_{1r}$ can be expressed as follows:

(A7) $$p_{1n}(\tau_{1n},\tau_{1r})=\tau_{1n}-\tau_{1n}\alpha +\tau_{1r}\alpha +\frac{(c-\tau_{1r})\delta }{2},p_{1r}(\tau_{1n},\tau_{1r})=s+\tau_{1r}\alpha +\frac{(c-\tau_{1r})\delta }{2}$$

Hence, the OEM’s optimal problem in the first period can be formulated as follows:

(A8) $$\prod^{S2*}=\underset{p_{1n},p_{1r}}{\max\;}\left((p_{1n}-c).D_{1n}+p_{1r}.D_{1r}\right)+\prod_{2}^{S2*}(\tau_{1r})$$

To ensure the objection function is concave in $\tau_{1n},\tau_{1r}$, we impose the condition of $-2+2\alpha +\frac{\lambda^{2}}{2\beta -2\beta^{2}}<0$ and $\frac{(3-2\delta )\lambda^{2}}{4\beta -4\beta^{2}}-(1-\alpha )(1-4\alpha +2\delta )>0$. The optimal value of $\tau_{1n},\tau_{1r}$ can be obtained from the first-order conditions, which can in turn be used to solve the equilibrium outcomes for the OEM in periods. Based on these, it is easy to prove Proposition 3. Proof of Corollaries 1–4 and Proof of Propositions 4–7. The results follow directly from the optimal results of the two settings under two scenarios. This completes the proof. □
